# ML‐UrineQuant: A machine learning program for identifying and quantifying mouse urine on absorbent paper

**DOI:** 10.14814/phy2.70243

**Published:** 2025-03-18

**Authors:** Warren G. Hill, Bryce MacIver, Gary A. Churchill, Mariana G. DeOliveira, Mark L. Zeidel, Marcelo Cicconet

**Affiliations:** ^1^ Laboratory of Voiding Dysfunction, Division of Nephrology, Department of Medicine Beth Israel Deaconess Medical Center & Harvard Medical School Boston Massachusetts USA; ^2^ The Jackson Laboratory Bar Harbor Maine USA; ^3^ Laboratory of Pharmacology Sao Francisco University Sao Paulo Brazil; ^4^ Image and Data Analysis Core, Department of Cell Biology Harvard Medical School Boston Massachusetts USA; ^5^ Present address: Deliberate Solutions Inc. New York New York USA

**Keywords:** artificial intelligence, mice, micturition, python, urology, void spot on paper, voiding dysfunction, VSA, VSOP

## Abstract

The void spot assay has gained popularity as a way of assessing functional bladder voiding parameters in mice, but analyzing the size and distribution of urine spot patterns on filter paper with software remains problematic due to inter‐laboratory differences in image contrast and resolution quality and non‐void artifacts. We have developed a machine learning algorithm based on Region‐based Convolutional Neural Networks (Mask‐RCNN) that was trained in object recognition to detect and quantitate urine spots across a broad range of sizes—ML‐UrineQuant. The model proved extremely accurate at identifying urine spots in a wide variety of illumination and contrast settings. The overwhelming advantage it offers over current algorithms will be to allow individual labs to fine‐tune the model on their specific images regardless of the image characteristics. This should be a valuable tool for anyone performing lower urinary tract research using mouse models.

## 1 INTRODUCTION

In lower urinary tract research using mice, a common technique for assessing bladder and urethral function is the void spot assay (VSA). This simple noninvasive assay involves placing mice in a cage with some form of absorbent paper on the cage floor, and after a period of time, recovering the paper to assess the size and distribution of urine spots (Bjorling et al., 2015; Chen et al., 2017; Hill et al., 2018; Luo et al., 2023; MacIver et al., 2023; Ruetten et al., 2023). The analysis is typically done after imaging the paper under ultraviolet light. While simple and easy to perform, the assay has proven to be remarkably useful for studying physiology and pathophysiology in disease models, genetically modified mice, and in drug and toxicology testing studies (Chen et al., 2020; Iguchi et al., 2023; Lavery et al., 2023; Oliveira et al., 2021). In particular, it has proven useful in longitudinal studies of bladder dysfunction with aging (Bartolone et al., 2020; Kim et al., 2020; MacIver et al., 2023).

One of the technical challenges that remains, however, is the analysis of the urine spot images obtained. Perhaps the most well‐known publicly available software application is *VoidWhizzard*, developed at the University of Wisconsin (Wegner et al., 2018) and made available as an ImageJ plugin. While some groups have found it useful, it has several drawbacks, including an inability to process certain image formats. The program relies on a thresholding step to separate the paper background from the brighter fluorescing urine. However, in order for this step to work it requires the images possess a number of specific features which, in practice, vary enormously between labs. First, the pixel intensity contrast between urine and paper needs to be substantial, and this can vary depending on factors such as (a) whether the mouse has a diuresis, for example, diabetic mice exhibit polyuria with the intensity of fluorescing substances in the urine diluted, (b) the type of paper used, which can be brighter or dimmer under UV light, and (c) the quality and wavelength of the UV illuminator used. If the contrast is inadequate, the thresholding step is either impossible or inaccurate. A second feature that interferes with simple thresholding is the fact that many UV illuminators do not illuminate the paper evenly, resulting in a background gradient that can be quite steep and is problematic for simple thresholding. The third important variable is image resolution, which needs to be reasonably high and can vary depending on the camera used and the file format generated.

These between‐lab variables are a problem for any software that relies on image features defined by pixel intensities. Therefore, we used a machine learning approach to develop a set of algorithms that can be trained on any set of images, regardless of their particular characteristics. We have called this Machine Learning UrineQuant (ML‐UrineQuant or MLUQ) By relying on segmentation of individual object instances, it is able to deliver superior spot detection and quantitation in an unbiased, rapid, high‐throughput manner.

## 2 METHODS

### 2.1 Ethical approval

Experiments performed on mice at BIDMC were done under approved IACUC protocol #0142021. Experiments performed by MGD were approved by the Ethics Committee on Animal Use of the University of Campinas (CEUA/UNICAMP, project number 582911). Experiments performed by GAC were performed under Jackson Lab IACUC protocol #06005.

### 2.2 Void spot assay

VSAs in our lab were performed as described previously (Hao et al., 2019; Kim et al., 2020; Xie et al., 2021). Mice are placed individually in empty mouse cages with pre‐cut filter paper (Blicks Cosmos blotting paper #10422‐1005) on the bottom. They are provided with food in the usual wire racks, but no water because water dripping on the filter paper creates dilution and spreading artifacts. Assays are run for 4 h, after which the mice are returned to their home cages and the filter paper is allowed to dry before being photographed under UV light (365 nm) in a Chromato‐Vue C75 imaging box with on board Canon camera (EOS Rebel T3–12 megapixels).

### 2.3 Image processing

**FIGURE 1:**
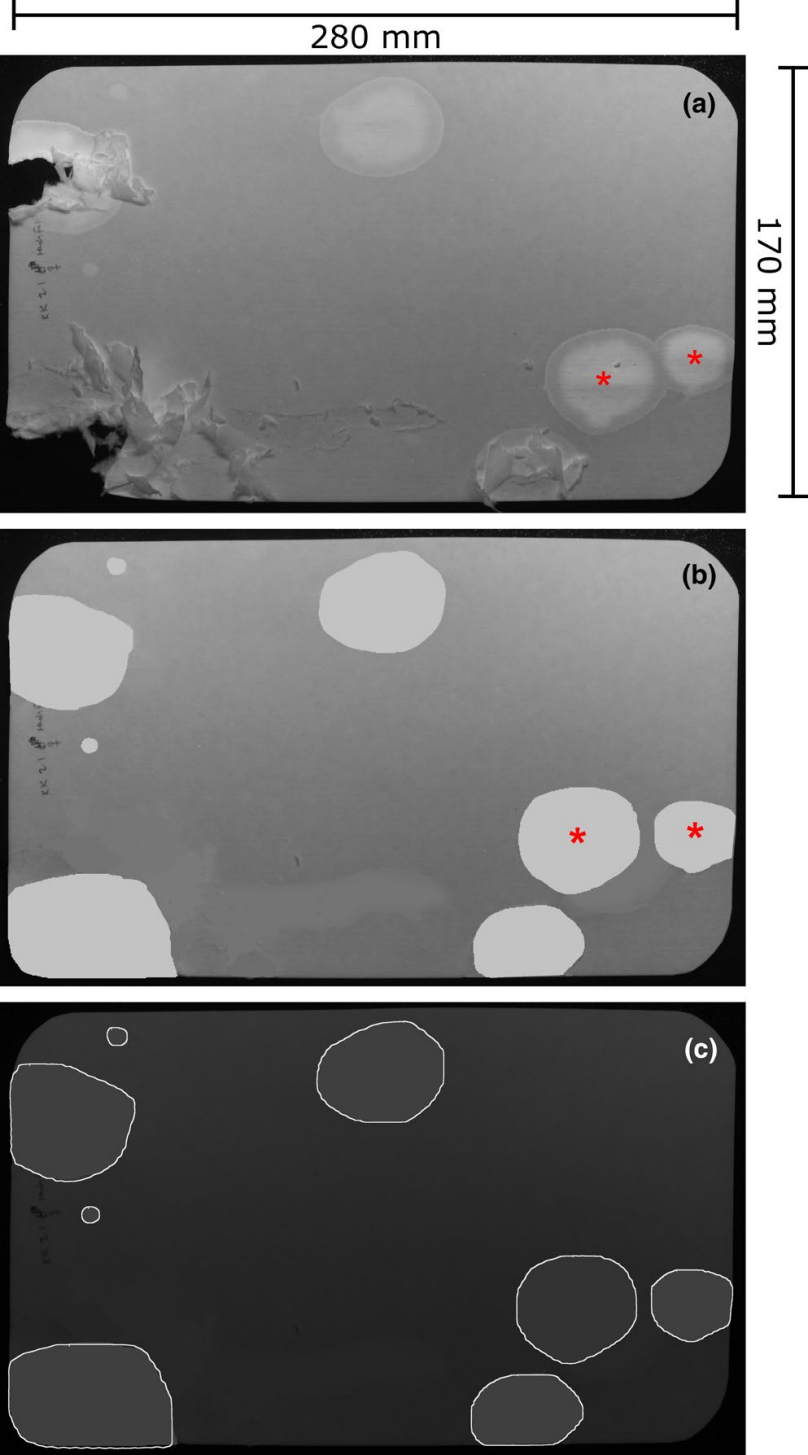
Common image artifacts require image processing and correction for accurate urine quantitation. (a) Example of a 4 h VSA filter paper photographed under UV light, with overlapping urine spots (red asterisks) and paper damage caused by a male TallyHo mouse. (b) The large overlapping spot was outlined using the freehand drawing tool, dragged to an empty part of the filter and filled with an appropriate color. The original spot was then filled in with background color. The damaged areas, in which the edges of the void are mostly visible were outlined and filled and torn folded edges corrected with background color. (c) ML‐UrineQuant detection of voids.

Prior to analysis with ML‐UrineQuant: Images should be exported in tif format and converted to 8‐bit grayscale. To ensure accurate spot counts and spot areas, overlapping spots are manually separated by outlining with the drawing tool in the Fiji version of ImageJ (https://imagej.net/software/fiji/downloads), and then copying and moving one of the spots to an empty area of the filter. Another issue frequently encountered is that mice sometimes chew and damage the filter paper, and this often occurs after a void, within the area covered by urine—since the paper is wet and soft. It is usually possible to see the fluorescent edges of the deposited urine, and it is a simple matter to outline and fill in the damaged area (see Figure 1).

The image analysis is primarily based on a machine learning model for instance segmentation developed in Python. More specifically, we fine‐tuned a Mask‐RCNN model [https://arxiv.org/abs/1703.06870], which was pretrained on the Common Objects in Context (COCO) dataset [https://cocodataset.org/#home]. We used the model architecture and pretrained weights provided by the PyTorch library [https://pytorch.org/tutorials/intermediate/torchvision_tutorial.html].

**FIGURE 2:**
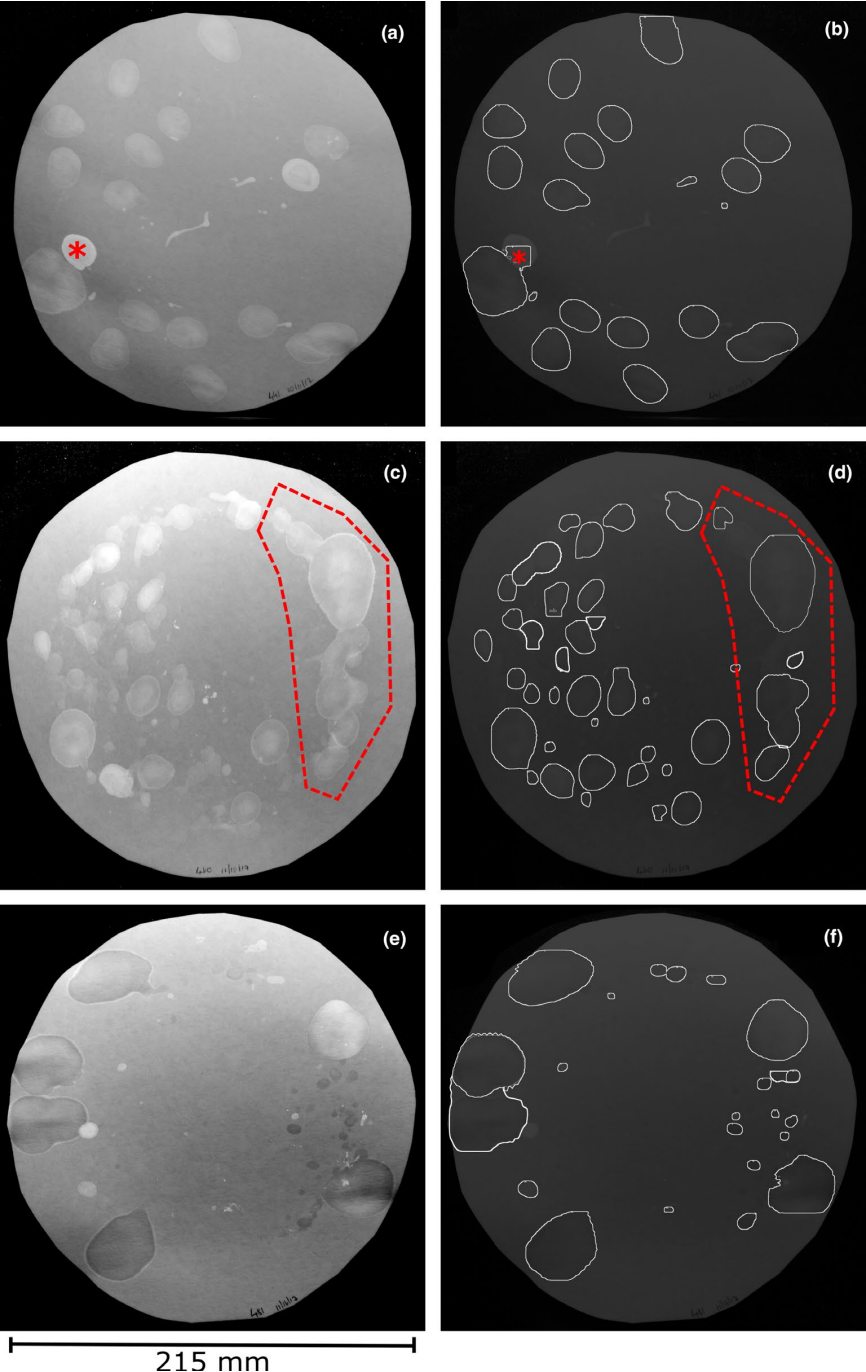
The model can be successfully trained on images with different characteristics. (a, c, e) Circular filters were cut to fit in metabolic cages and then mice undergoing awake cystometry 7 days after intrabladder catheter implantation, were free to move around the cage and void spontaneously for 60 min. Saline was continuously infused at 25 μL/min. After training on 21 images the model was deployed and object detection results are shown in (b), (d), and (f).

Sixty‐two images of size 2136 × 1424 pixels were annotated to fine‐tune the model, and we wrote custom code to deploy the model on larger images. Specifically, our 12.2 MB tif files were split into four, and then a number of the “split” images were randomly selected to create the training set. Each image was then annotated in Fiji using the drawing tool to outline urine spots, which were added to the ROI manager and saved. For the circular metabolic cage filters shown in Figure 2, the model was trained on 21 images. Following deployment on the larger images, the model produces .png and .csv files as outputs, which show the voids detected and the area of each void in pixels. Each void is uniquely identified by number. Access to the software package may be found at https://doi.org/10.6084/m9.figshare.27609420.v1.

## 3 RESULTS

Figure 1a shows the 4‐h void spot pattern of a 5‐month‐old TallyHo male mouse (The Jackson Laboratory, Maine, U.S.A; strain #005314). Several features are notable. TallyHo's are hyperglycemic, and one of the micturition characteristics of this strain that we described recently is that they exhibit a pronounced polyuria (MacIver et al., 2023). Consequently, the number of voiding events and the size of each void are much larger than is typically seen with C57BL/6J mice. From an image analysis perspective, there are two problems with attempting urine quantitation. First, the overlapping spots at right (red asterisks) and the chewed and damaged edges at left, both of which occur at urine‐soaked edges. Figure 1b shows that following image correction using Fiji, the overlapping spots are separated and the damaged void spots are outlined and “filled” using the “color picker” tool. Deployment of the ML‐UrineQuant model on the “corrected” image results in the detection of voids, as shown in Figure 1c.

**FIGURE 3:**
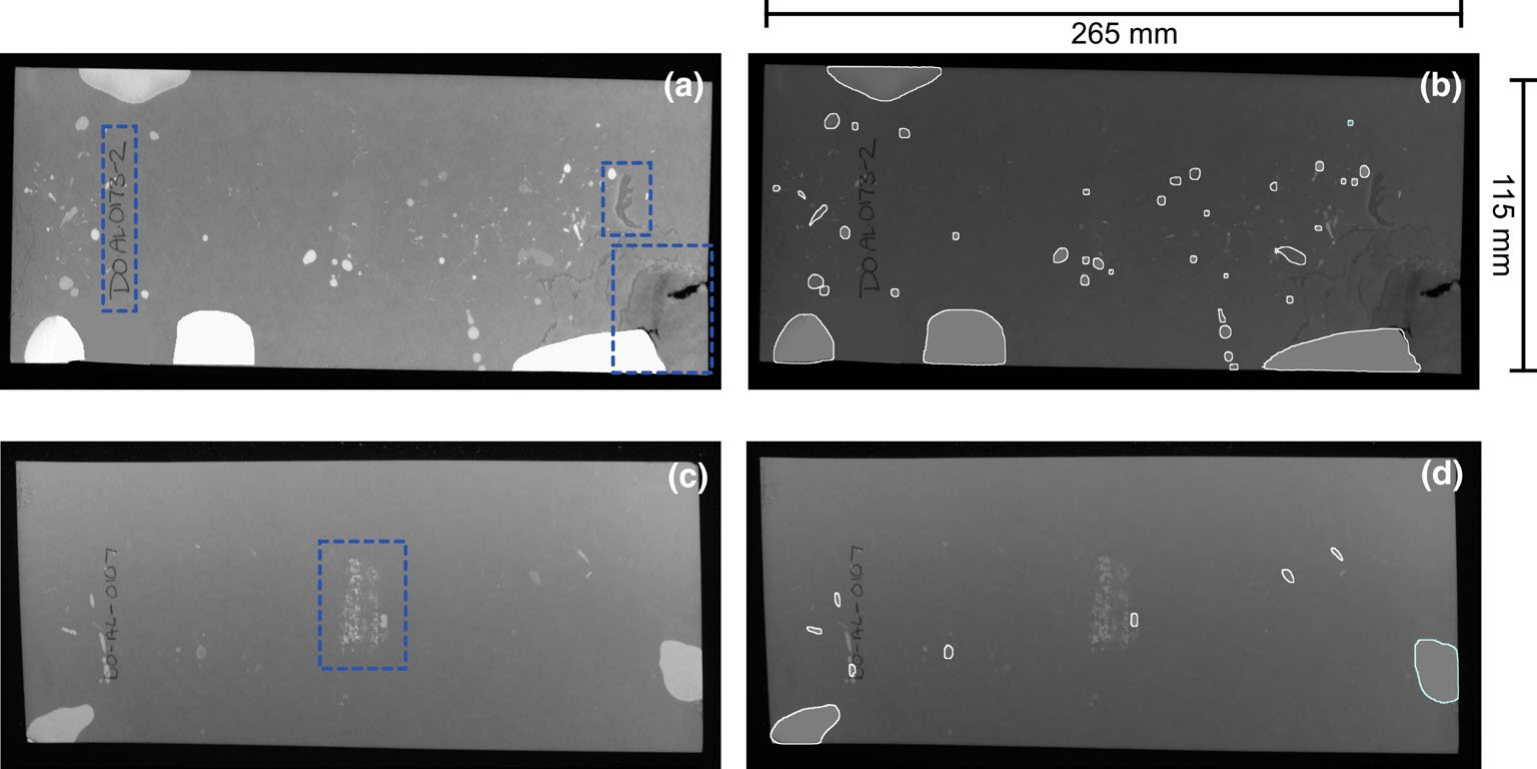
Visible image artifacts such as writing, scratches and tearing are ignored by ML‐UrineQuant. (a, c) UV photographs of 4 h VSA filter papers with highlighted artifacts in blue dashed boxes. (b, d) results of model deployment on (a) and (c).

Figure 3 illustrates the power of the model to ignore image artifacts that do not conform to the model's learning of what constitutes a void. Figure 3a shows dashed blue boxes around image features that are not voids (writing and filter paper damage) and 3FB shows the voids detected. In Figure 3c the area in the center that is outlined, is the result of an identification sticker placed on the underside of the paper. The glue that bled through fluoresces, and could be problematic for software that is focused on pixel intensities. Since it doesn't have the shape or pattern the model was trained to recognize, it is ignored (Figure 3d). Therefore, the accuracy of the model at distinguishing and detecting only urine spots is extremely good.

**FIGURE 4:**
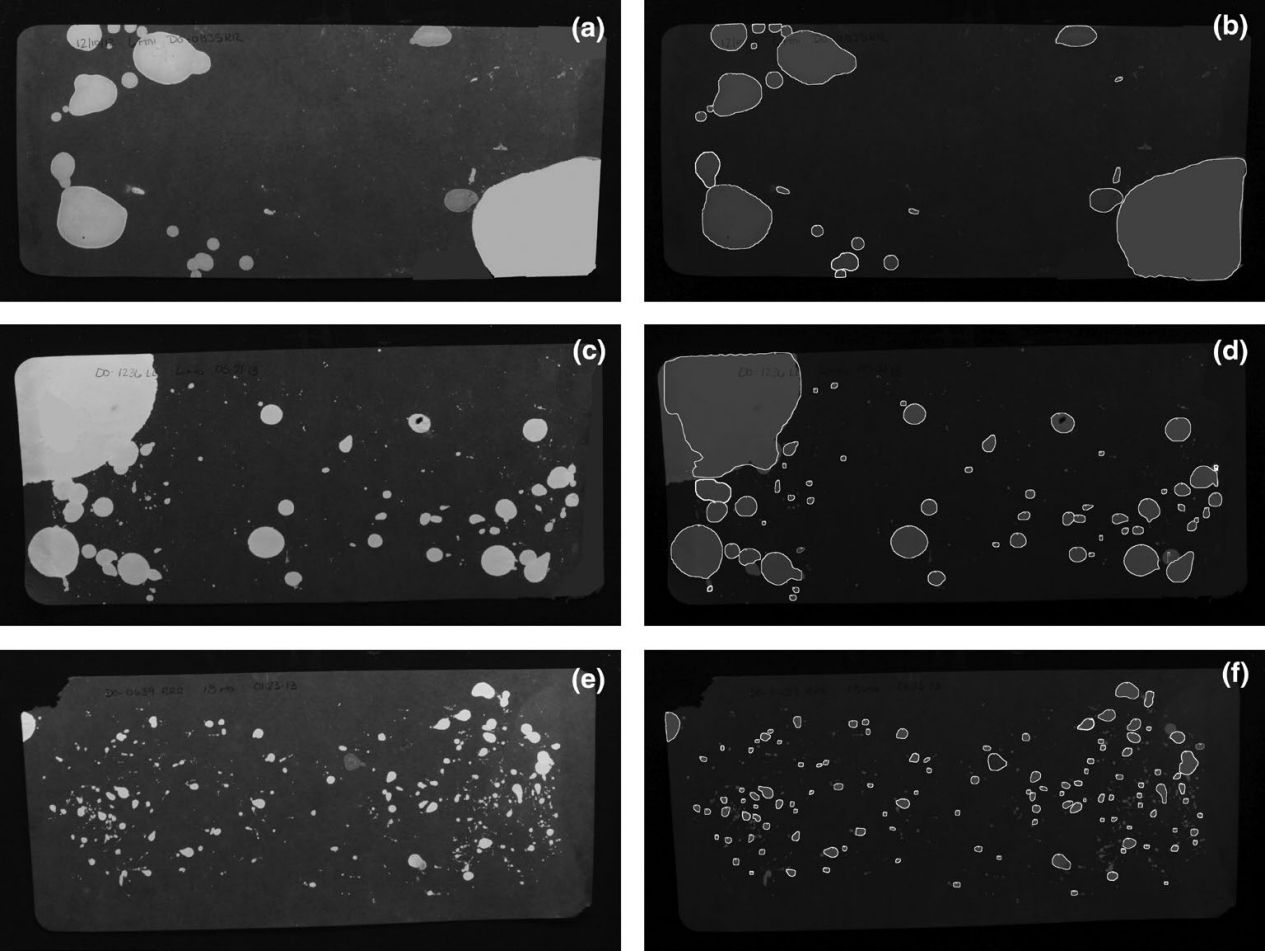
Using different filter papers provides different image characteristics for the model to contend with. (a, c, e) 4 h VSA images of Whatman #1 filter paper. (b, d, f) results of model deployment on (a), (c), and (e).

The Blicks Cosmos blotting paper we routinely use (Figures 1 and 3) has the advantage that it is thicker than regular filter paper and is therefore tougher to tear and chew, and also restricts the diameter of urine spread. By making the void areas smaller, it reduces the number of overlapping spots. It does, however, suffer from the drawback of being brighter under UV light, thus reducing the contrast with urine. Figure 4 shows void spot patterns of three 6‐month‐old female mice from the JAX diversity outcross population (Svenson et al., 2012) (Figure 4a,c,e). These mice were tested on Whatman #1 filter paper where the improved contrast between urine and background is immediately apparent (Figure 4a,c,e). The primary voids seen in Figure 4a,b also illustrate how much further the urine spreads as the paper is very thin. Without further or specific training of the model on these images to account for the different characteristics, it was still highly efficient at spot detection (Figure 4b,d,f), with the exception of the large void with several overlapping smaller spots in Figure 4d. When this occurs, it is usually due to the presence of other very near and overlapping spots. This can be fixed by editing the image to move the overlapping spots to a different part of the filter paper and re‐running the program.

**FIGURE 5:**
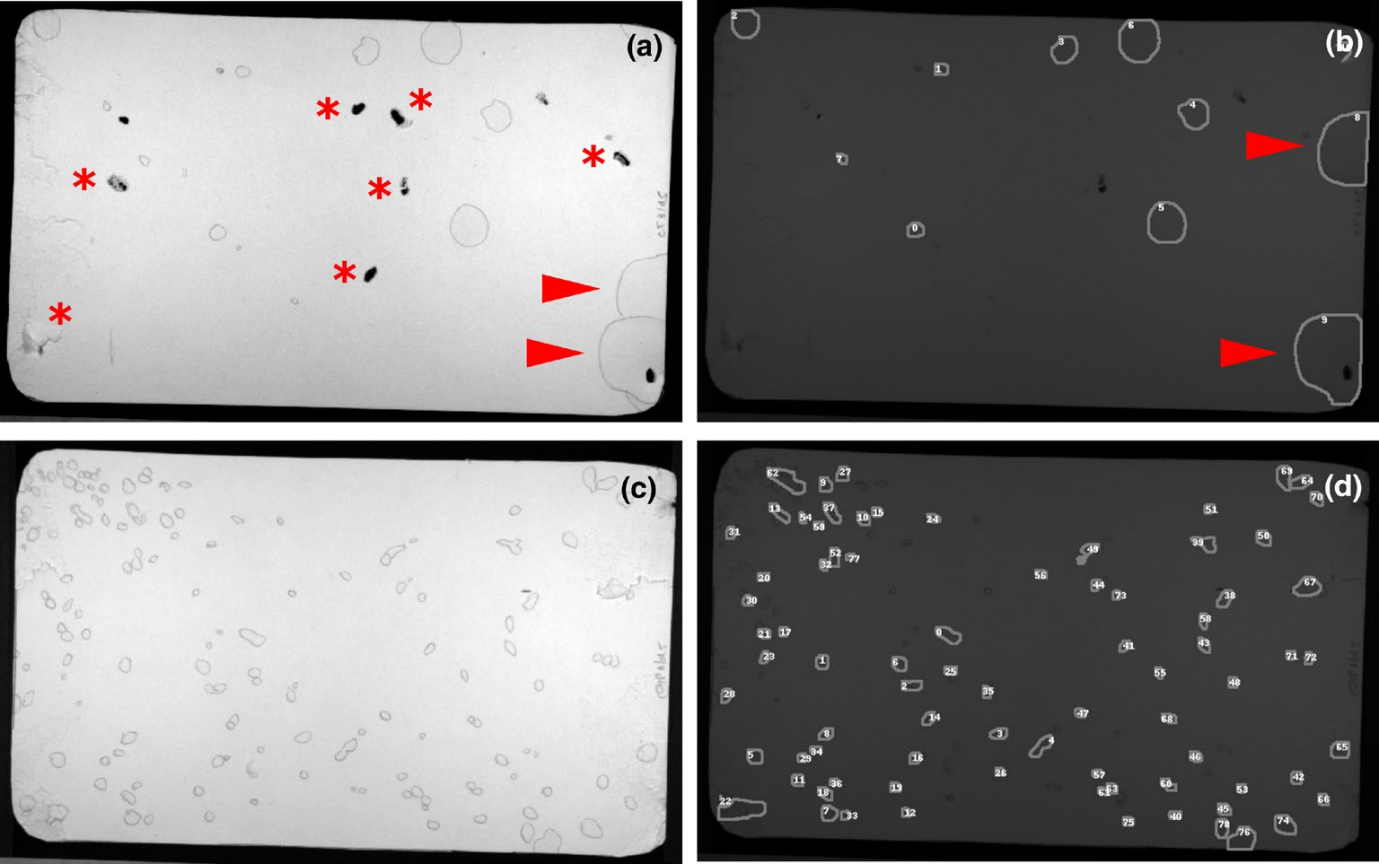
ML‐UrineQuant recognizes objects with very different illumination settings even without further training. Left panels) Void spot papers under visible light in which urine spots were manually outlined in pencil. Asterisks indicate image artifacts; arrowheads point at overlapping voids. Right panels) results of model deployment on images at left.

Figure 5 further illustrates the power of the model to accurately segment images with very different contrast characteristics. In this case, the laboratory performing the VSA did not have a UV illuminator and so simply outlined the spots in pencil. These are representative VSA filter papers taken from a study of cyclophosphamide‐induced cystitis, with a control filter shown in the upper left and a mouse injected with 300 mg/kg cyclophosphamide at the bottom left (de Oliveira et al., 2022). The presence of incontinence in response to bladder pain is clear. In the control image, overlapping spots (arrowheads) were separated using Fiji, and the presence of small black artifacts and torn paper (asterisks) did not segment when the model was deployed.

In another study on the effect of traumatic brain injury (TBI) on voiding dysfunction, mice were subjected to awake, conscious, freely moving cystometrograms, in metabolic cages (Albayram et al., 2019). As shown in Figure 2, circular Blick's Cosmos blotting paper was placed on the bottom of the cage. Mice were infused continuously with saline for 60 min into the bladder (25 μL/min) from an in‐dwelling catheter exteriorized at the back of the neck. The spot patterns obtained from different groups of animals seen in Figure 2a,c,e are notable firstly, because the amount of urine and number of voiding events is much greater than for a normal VSA (due to the supraphysiological filling rate), and secondly, because of saline dilution, the urine spots vary greatly in their brightness with some hardly fluorescing at all. When deployed on these images, after being trained on a subset of them, the results were very good. In Figure 2a, the segmented image that resulted (Figure 2b) has one poorly defined overlapping spot marked with an asterisk. Figure 2c shows the filter paper of a TBI mouse at 8 months after injury. No image correction to separate overlapping spots was performed on any of these, and the area defined by the red dashed polygon shows multiple continuous overlapping urination events with low contrast. Despite this the resulting segmentation is, while not perfect, still quite good (Figure 2d). The third example shown in Figure 2e is unusual in that many of the spots, both large and small have an “inverse” coloration, that is, rather than appearing brighter they are darker. The reason for this is unclear. However, the objects or instances corresponding to voids are detected and segmented well. If greater accuracy in void size and volume were required the two large voids at left could be separated using Fiji.

**FIGURE 6:**
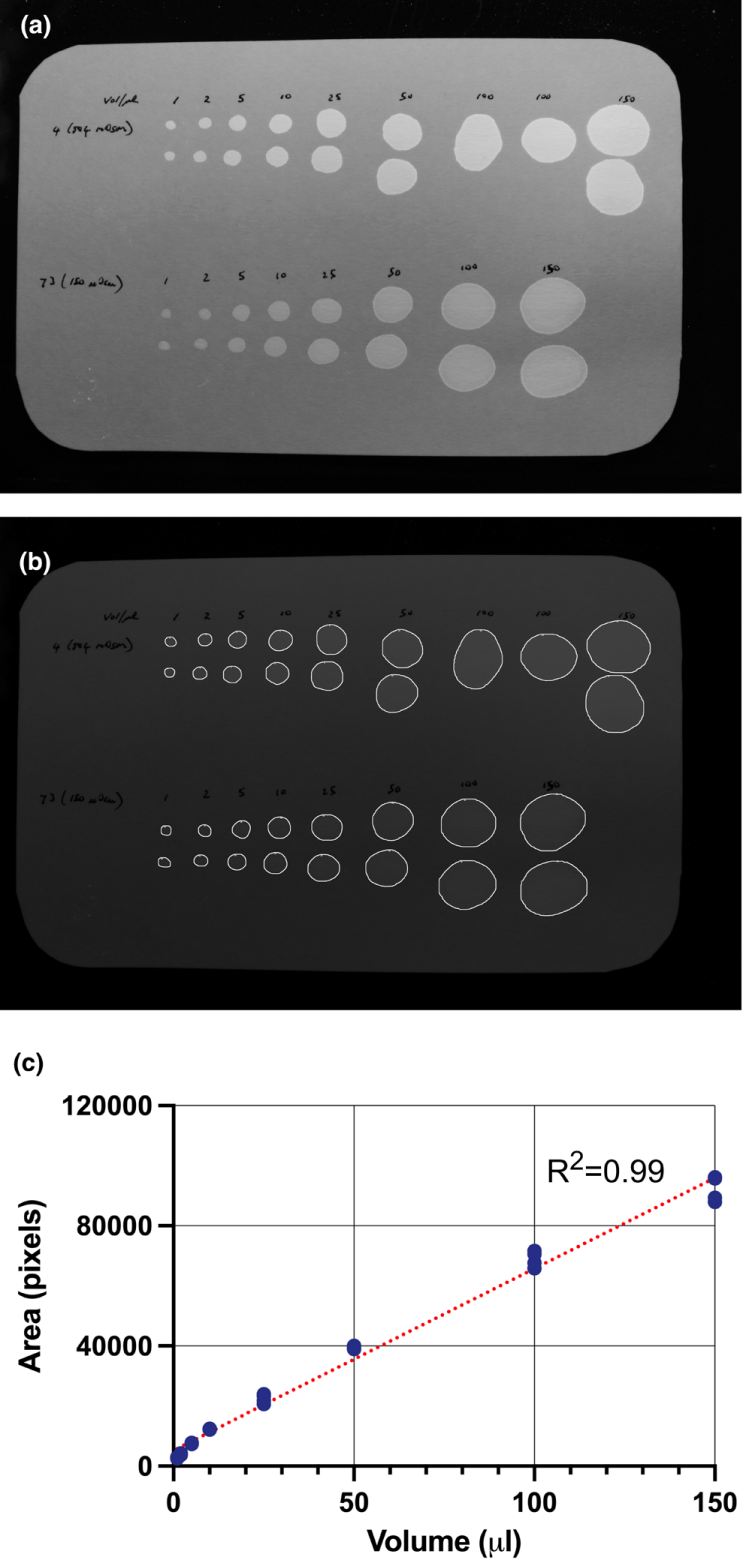
Mouse urine of different osmolalities (504 and 150 mOsm/kg) was spotted onto Blicks filter paper at volumes of 1, 2, 5, 10, 25, 50, 100, and 150 μL in duplicate. (a) photograph taken under UV light; (b) the segmentation result produced by MLUQ; (c) the area:Volume standard curve showing linear regression analysis.

The model was trained on images for which the ground truth was defined on spots that ranged in volume from <1 μL to ~500 μL. As such, the sensitivity of detection is approximately 1 μL. Figure 6 shows the area:volume relationship of urine spots on Blicks blotting paper, which clearly shows that urine volumes of 1 μL are easily detected and that the area:volume relationship is linear. Figure 6a shows urine of different osmolalities from two different mice, pipetted in duplicate over a range of volumes. The model output is shown in Figure 6b, and the standard curve is shown in Figure 6c. The upper set of spots was urine with an osmolality of 504 mOsm/kg, while the lower set was 150 mOsm/kg. All of the data is shown plotted on the standard curve and demonstrate that the different osmolalities did not affect the diffusive spread.

**FIGURE 7:**
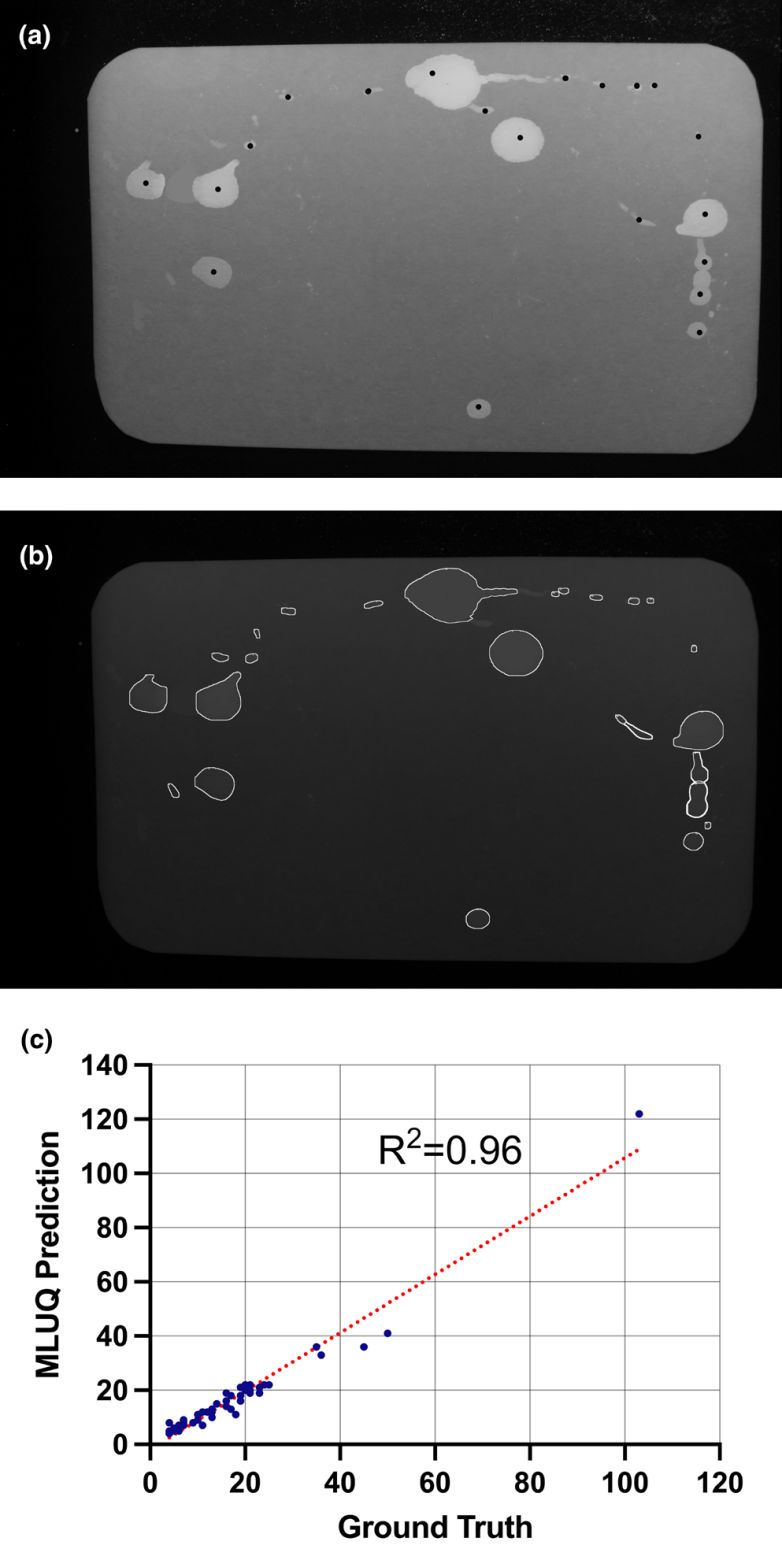
Linear regression analysis on 48 filter paper images produced by individual male and female TallyHo and KK‐A^y^ mice. (a) urine spots defined by us as ‘ground truth’ were marked with black dots; (b) the model's prediction of urine deposits; (c) the correlation between prediction and ground truth for all 48 images. Discrepancies are mostly on very small marks that are typically 1 μL or less.

To arrive at an estimate of the model's precision we took 48 filter images from a recent study we performed, on two strains of mice with type 2 diabetes (KK‐A^y^ and TallyHo) (MacIver et al., 2023). There were 24 mice of each strain, and 12 males and 12 females of each. We then defined the “ground truth” for each image in each set by visually identifying and counting every spot that we felt the model should be able to detect. For large and medium sized spots this is easy, but on images with large numbers of very small spots and marks, the definition (even to the investigator) becomes somewhat arbitrary. We performed a linear regression analysis to determine how well correlated our ground truth definitions were to the model's predictions. Figure 7c shows the result, which gave a goodness of fit, R squared value of 0.956. Deviations from the ground truth are most pronounced on filters with large numbers of very small spots and marks; however, with a slope of 1.075, the ground truth and model predictions are very well synchronized. The image shown in Figure 7a is an example of a 4‐h void spot assay, with the ground truth spots selected by us indicated as black dots. The MLUQ prediction is shown in Figure 7b. There are a few very small marks that we did not consider “urination events” and did not mark as such, but which the model detected and counted.

MLUQ outputs the results into a user‐defined folder, as a png image showing the segmentation and a CSV file containing two columns; an i.d. column in which each spot is given a number and an area column with the number of pixels in each spot. To extract useful aggregate information on each filter paper, we wrote an additional piece of software in the R programming language called “Fil3” that reads in the png and csv files, segregates background pixels from filter paper pixels, sums the number of spots, converts pixel areas to volumes based on a standard curve similar to that shown in Figure 6c, separates the primary and microvoids, and calculates the means. The results from a folder containing matching png and csv files is a spreadsheet titled VoidDataSummary.csv as shown in Table 1 that gets written into the same folder. This software is available for download at https://doi.org/10.6084/m9.figshare.28232822.v2.

**TABLE 1:** Fil3 analysis on a folder containing MLUQ data from 10 filter paper images. The data on urine spots for each filter is separated into two volume bins, with primary voids being those >20 μL and Micro Voids being those between 1 and 20 μL.

Image	Total_Volume (μl)	Total_Spot_No.	Primary_Voids	Primary_Voids_Mean_Volume (μl)	Micro_Voids
IMG_8945_areas.csv	278.2	3	3	92.7	0
IMG_8946_areas.csv	151.6	6	1	128.1	5
IMG_8947_areas.csv	196.5	3	2	97.2	1
IMG_8948_areas.csv	327.7	5	2	154.2	3
IMG_8949_areas.csv	472.4	2	2	236.2	0
IMG_8950_areas.csv	490.9	7	2	238.7	5
IMG_8951_areas.csv	758.6	21	11	65.1	10
IMG_8952_areas.csv	251.3	14	1	197.4	13
IMG_8953_areas.csv	176	7	2	79.3	5
IMG_8954_areas.csv	424.9	64	1	32.6	63

## 4 DISCUSSION

The “sensitivity” of the model depends on the ground truth on which it was trained. In our case, we trained the algorithm to detect a wide range of deposited volumes from ~1 μL to 1000 μL. There is no reason it could not be trained to accurately detect and quantify smaller spot areas, but for practical purposes, there is little physiological meaning to spots of <1 μL. Indeed, they are more likely to be artifacts of claw marks or fur contamination. Therefore, we conclude that it possesses excellent sensitivity, which should be more than sufficient for the needs of most investigators.

The question of the model's accuracy is a tough one for several reasons. One is that void spotting images can be very messy, and particularly in instances where the mouse has significantly chewed and/or damaged the paper. Another example of hard to analyze filters can occur in some strains when the objective is to quantitate male voiding parameters. Males can exhibit dominance, territoriality, and “marking” behavior, where urination is used to delineate ownership of a space. In some cases, we have seen males leave hundreds of small streaky spots that often overlap prodigiously. Accuracy in differentiating individual events in a case like this is obviously much poorer. Another take is in how to define accuracy. Should this be in terms of identifying “all” spots and not missing any, or should it be in terms of the accuracy in identifying void versus non‐void visual artifacts ? As seen in Figure 7, the ability of MLUQ to count spots according to predefined ground truth is very good, with an R squared value of 0.956. Where it excels, however, is in distinguishing void from non‐void objects, even in images with low contrast levels.

While we believe the software is versatile enough to be helpful to any laboratory, it does have some weaknesses. Overlapping spots present a problem that this model is, at present, unable to solve. While it can sometimes identify spots which intersect, it appears to be a matter of degree. Barely overlapping spots are often able to be separately identified. Examples of this can be seen in Figure 4c,d on the lower left side. However, on the same filter, the multiple overlaps of very differently sized spots (top left corner) appear to confuse it somewhat. A similar phenomenon occurs in Figure 2c,d, in the dashed outline region. This is clearly a messy pattern from an object discrimination standpoint, and the model is only partially successful. In practice, these problems can be overcome with some manual editing in Fiji and then rerunning the program. While not ideal because of the extra time required, it does allow a work around.

This freely available tool should improve the ability of labs with very different protocols, filter paper choices, mouse models, and imaging setups to accurately quantitate and analyze their results in an unbiased and rapid manner. It also facilitates and automates the analysis of large data sets for high‐throughput purposes. Its utility in our lab was seen in a large‐scale longevity study of 970 Diversity Outcross mice, in which we quantitated void spot assays from this cohort multiple times over their lifespan. In total, we analyzed 3394 filter papers (Di Francesco et al., 2024), an effort that would have been impractical with Fiji. We conclude this is a significant improvement on currently available software tools, and because it is open source, it also provides a platform to investigators and developers for further improvement and refinement.

## AUTHOR CONTRIBUTIONS

WHG conceived the work, acquired and analyzed data, and drafted the manuscript; BM acquired data and drafted, and edited the manuscript; GAC acquired data and edited the manuscript; MGD acquired data and edited the manuscript; MLZ contributed intellectually to the design and edited the manuscript; MC designed and refined the algorithm and edited the manuscript. All authors approved the final version of the manuscript; agree to be accountable for all aspects of the work in ensuring that questions related to the accuracy or integrity of any part of the work are appropriately investigated and resolved, and qualify for authorship.

## FUNDING INFORMATION

We wish to acknowledge funding from the JAX Nathan Shock Center P30AG038070 and from NIDDK P20DK097818.

## Data Availability

Data consists of examples of the model's capabilities. Figures 1, 2 and 5 show void spot images that were part of published studies that have been cited in Results.
